# HIV-Associated Disruption of Tight and Adherens Junctions of Oral Epithelial Cells Facilitates HSV-1 Infection and Spread

**DOI:** 10.1371/journal.pone.0088803

**Published:** 2014-02-21

**Authors:** Irna Sufiawati, Sharof M. Tugizov

**Affiliations:** 1 Department of Medicine, University of California San Francisco, San Francisco, California, United States of America; 2 Department of Orofacial Sciences, University of California San Francisco, San Francisco, California, United States of America; University of Pennsylvania School of Veterinary Medicine, United States of America

## Abstract

Herpes simplex virus (HSV) types 1 and 2 are the most common opportunistic infections in HIV/AIDS. In these immunocompromised individuals, HSV-1 reactivates and replicates in oral epithelium, leading to oral disorders such as ulcers, gingivitis, and necrotic lesions. Although the increased risk of HSV infection may be mediated in part by HIV-induced immune dysfunction, direct or indirect interactions of HIV and HSV at the molecular level may also play a role. In this report we show that prolonged interaction of the HIV proteins tat and gp120 and cell-free HIV virions with polarized oral epithelial cells leads to disruption of tight and adherens junctions of epithelial cells through the mitogen-activated protein kinase signaling pathway. HIV-induced disruption of oral epithelial junctions facilitates HSV-1 paracellular spread between the epithelial cells. Furthermore, HIV-associated disruption of adherens junctions exposes sequestered nectin-1, an adhesion protein and critical receptor for HSV envelope glycoprotein D (gD). Exposure of nectin-1 facilitates binding of HSV-1 gD, which substantially increases HSV-1 infection of epithelial cells with disrupted junctions over that of cells with intact junctions. Exposed nectin-1 from disrupted adherens junctions also increases the cell-to-cell spread of HSV-1 from infected to uninfected oral epithelial cells. Antibodies to nectin-1 and HSV-1 gD substantially reduce HSV-1 infection and cell-to-cell spread, indicating that HIV-promoted HSV infection and spread are mediated by the interaction of HSV gD with HIV-exposed nectin-1. Our data suggest that HIV-associated disruption of oral epithelial junctions may potentiate HSV-1 infection and its paracellular and cell-to-cell spread within the oral mucosal epithelium. This could be one of the possible mechanisms of rapid development of HSV-associated oral lesions in HIV-infected individuals.

## Introduction

Herpes simplex virus type 1 (HSV-1) is a common oral pathogen that causes multiple oral disorders such as ulcers, necrotic lesions, and gingivostomatitis. Oral epithelium is also infected with HSV-2 [1], but to a lesser extent. HIV infection leads to reactivation and spread of herpesviruses, including HSV-1 and -2, in oral and genital mucosa [2], [3], [4], [5], [6], [7], [8], [9], [10]. HIV infection causes attenuation of the immune system by substantially depleting CD4^+^ T cells in peripheral blood, lymphoid organs, and mucosal tissues, leading to CD8^+^ T cell dysfunction [11], [12], [13]. HIV-mediated depletion and dysfunction of CD4^+^/CD8^+^ T immune cells can lead to the activation of herpesviruses [2], [3], [4], [5], [6], which are usually latent under normal immune surveillance [14].

In addition to attenuation of the immune system, HIV infection can impair the barrier function of various mucosal epithelia, including oral, intestinal and anogenital mucosa [15], [16], [17], [18], [19], [20]. This in turn may facilitate the spread of opportunistic infections, including HSV-1/2, throughout the epithelium. HIV tat and gp120 proteins play an important role in the impairment of the mucosal barrier by disrupting epithelial tight junctions (TJs). HIV tat and gp120 are transactivator and envelope proteins that activate multiple signaling pathways, including mitogen-activated protein kinase (MAPK) signaling, which lead to disruption of TJs through aberrant internalization of TJ proteins and their down-regulation and/or proteasome-mediated degradation [21], [22], [23], [24], [25], [26], [27], [28], [29], [30], [31], [32].

Nectin-1 is a poliovirus receptor-related protein 1 (PRR1/HveC/CD111) and a Ca2+-independent cell adhesion protein of the immunoglobulin superfamily [33], [34]. Nectin-1 binds to HSV glycoprotein D (gD), facilitating entry of virions into epithelial cells and cell-to-cell spread of progeny virions [35], [36], [37], [38], [39], [40], [41], [42]. Nectin-1 is sequestered in the intercellular junctions, limiting the access of HSV [43].

In this study we wanted to explore the role of HIV-associated disruption of oral mucosal epithelium in HSV-1 infection and spread by using polarized oral keratinocytes as a model system. Our data show that HIV tat and gp120 proteins disrupt oral epithelial TJs and adherens junctions (AJs), leading to the paracellular spread of HSV, which may lead to rapid dissemination of virus within the mucosal environment and to saliva, increasing the risk of spreading viral infection to others. HIV tat/gp120-induced disruption of AJs exposes nectin-1 for HSV-1 binding. Furthermore, HIV-associated disruption of AJs and exposure of nectin-1 promote HSV-1 infection and cell-to-cell spread of the virus, leading to the rapid progression of HSV-caused mucosal lesions and ulcers.

## Materials and Methods

### Ethics Statement

This study was conducted according to the principles expressed in the Declaration of Helsinki. The study was approved by the Committee on Human Research of the University of California–San Francisco (IRB approval #: H8597-30664-03). All subjects provided written informed consent for the collection of samples and subsequent analysis.

### Establishment of Polarized Oral Epithelial Cells

To establish polarized cells from primary oral epithelial cells, we propagated primary keratinocytes from adult tonsil tissue samples, as described in our previous work [44], [45]. Tonsil epithelial cell lines were grown in culture medium SAGM (Lonza Inc., Allendale, NJ) and incubated at 37°C in a humidified incubator containing 5% CO_2_. Polarized cells were established in 0.4-µm Transwell two-chamber filter inserts (12-well inserts) as described previously [45], [46], [47]. The polarity of epithelial cells was confirmed by immunodetection of TJ protein zonula occludens-1 (ZO-1), and measurement of transepithelial resistance (TER) and paracellular permeability [45]. TER was measured with an epithelial Millicell-ERS volt-ohm-meter (Millipore Corp., Billerica, MA). Paracellular permeability was evaluated by adding horseradish peroxidase (HRP)-conjugated goat anti-donkey IgG (Fab’)_2_ (Jackson ImmunoResearch Laboratories, Inc., West Grove, PA) to the upper filter compartment and photometrically assaying the medium from the lower compartment for HRP with *o*-phenylenediamine dihydrochloride as the substrate [45], [48]. Detection of IgG in the lower chamber indicated leakage of IgG from the upper chamber.

### Viruses and Viral Proteins

HIV-1_SF33_ dual-tropic virus was propagated in peripheral blood mononuclear cells as described [46], [49]. HIV was inactivated by UV irradiation at 100 mJ/cm^2^ [50]. The virus stocks were grown and quantitated for p24 antigen using a p24 ELISA assay and stored in aliquots at 80°C. Recombinant HIV-1 (Bal strain) wild-type tat and inactive mutant tat proteins were purchased from ImmunoDX, LLC (Woburn, MA). Mutant tat was generated by substitution of the basic arginine-rich domain at 49–57 aa and the integrin-binding RGD motif in the C terminus with alanines. HIV-1 (Bal strain) gp120 was provided by the NIH AIDS Research Reagent Program. gp120 was inactivated by incubation at 85°C for 30 min [24]. All proteins were stored at –80°C in the dark before use.

HSV-1 (strain F) was grown and titered in Vero cells [51]. The truncated form of HSV-1 gD ectodomain gD(306t), which contains 1–306 residues of the ectodomain and lacks transmembrane and cytoplasmic domains [52], was provided by Gary H. Cohen and Roselyn J. Eisenberg (Department of Microbiology, School of Dental Medicine, University of Pennsylvania, Philadelphia, PA). HSV-1 gD(306t) was produced in the baculovirus system [52].

### Treatment of Polarized Cells with Cell-free HIV Virions, HIV Proteins tat and gp120, and HSV gD

Infectious or UV-inactivated HIV-1_SF33_ was added to the apical surface of polarized epithelial cells at 20 ng/ml of p24 antigen. Active and inactive recombinant tat and/or gp120 (10 ng/ml of each) were added to polarized tonsil cells from both apical and basolateral surfaces. HSV-1 gD (306t) at 10 ng/ml was added to polarized cells from both surfaces. Culture medium was changed daily to add fresh virus or proteins. Disruption of TJs and AJs was examined by immunostaining of their marker proteins ZO-1 and E-cadherin, respectively. Expression and localization of ZO-1 and E-cadherin as a ring shape was considered an intact junction. Diffuse, dot-like patterns in the cytoplasm were considered disrupted junctions [15]. Disruption of epithelial junctions was also monitored by measuring TER and paracellular permeability [46], [47].

### Immunofluorescence Assay

Polarized epithelial monolayers were fixed with 4% paraformaldehyde for 20 min, permeabilized with 0.05% Triton X-100, and washed three times with PBS. To determine disruption of TJs, we immunostained cells with mouse monoclonal antibodies against ZO-1 (Invitrogen/Life Technologies, Austin, TX) for 1 h at room temperature. For detection of AJs, polarized cells were stained with rabbit antibodies to E-cadherin and mouse monoclonal antibodies to nectin-1 (both from Invitrogen/Life Technologies). To determine HSV-1 infection, we immunostained infected cells with mouse antibodies against HSV-1 ICP4 and gB (both from Santa Cruz Biotechnology, Inc., Dallas, TX), rabbit anti-gD (HSV-1) (MyBioSource, San Diego, CA), and goat anti-HSV-1/2 antiserum (AbD Serotec, Raleigh, NC). All antibodies were diluted 1∶50 in blocking solution (3% BSA and 0.03% saponin in PBS). Monolayers were washed and then incubated for 25 min with FITC- or TRITC-labeled secondary antibodies (Vector Laboratories, Inc., Burlingame, CA). Cell nuclei were counterstained with TO-PRO-3 (blue) or propidium iodide (red) (Molecular Probes/Life Technologies). Cells were analyzed by using a Leica SP5 laser confocal microscope.

### Assay for HSV-1 Spread through a Paracellular Route of Disrupted Polarized Oral Epithelial Cells

For the HSV-1 paracellular spread assay, HSV-1 at a multiplicity of infection (MOI) of 10 PFU per cell was added to the apical surface of polarized monolayers, and cells were incubated at 37°C for 1, 2, or 4 h. At the various time points, culture medium was collected from the basolateral chamber of Transwell inserts and used to infect Vero cells, which were grown on cover slips on 24-well plates. After 4 h, cells were fixed for the immunofluorescence assay. HSV infection was quantified by detection of HSV-1 ICP4 protein, and the percentage of infected Vero cells was determined.

### HSV Infection and Cell-to-Cell Spread in Polarized Tonsil Epithelial Cells

To examine HSV-1 infection in polarized tonsil epithelial cells, we added HSV-1 to apical and basolateral membranes at an MOI of 4 and 40 PFU per cell, respectively. This resulted in the equal infection of virus at approximately 4 virions per cell from both membranes, because about 90% of basolaterally added virions could be trapped in the filter pores, and about 10% of virions reach the basolateral membranes [45], [47], [53]. Cells were cultured for 24 h and fixed and immunostained for HSV-1 gB using mouse monoclonal antibodies (Santa Cruz Biotechnology).

To examine the role of nectin-1 in HSV-1 infection, we preincubated polarized cells with mouse monoclonal antibody to nectin-1 (clone CK-8; Invitrogen/Life Technologies) at 10 µg/ml for 1 h, and cells were then infected with HSV-1. After 24 h, cells were fixed, and HSV infection was evaluated by immunostaining of cells with polyclonal goat anti-HSV-1/2 antibodies (AbD Serotec). HSV infection was quantified by counting infected cells, and the percentage of infected cells was determined.

To determine cell-to-cell spread of HSV-1 in polarized epithelial cells, a viral plaque assay was used [47]. Polarized cells were infected with HSV-1 from the basolateral surface at an MOI of 0.01 PFU per cell, and cells were incubated for 2 h at 37°C. The cells were then washed once with serum-free medium and overlaid with serum-free medium containing 0.5% methylcellulose from apical and basolateral chambers; this prevents the spread of released virions but allows cell-to-cell spread of progeny virions. Cells were cultured for 3 days. To detect HSV-1-infected viral foci or plaques, cells were fixed and immunostained with goat polyclonal anti-HSV-1 antibodies (AbD Serotec). The number of viral plaques was counted on a minimum of 3 filter inserts for each experiment, and average plaque numbers were expressed per insert. HSV-1 cell-to-cell spread was evaluated by quantitative analysis of HSV-1-infected plaques. Foci containing 5 or more infected cells were considered plaques, and cell numbers in each plaque were counted. A minimum of 30 plaques for each experimental condition were evaluated and the average number of cells per plaque was expressed.

For inhibition of HSV-1 cell-to-cell spread after 2 h of infection, cells were washed, and culture medium with antibodies to nectin-1 (Invitrogen/Life Technologies) and/or HSV-1 gD (MyBioSource, San Diego, CA) (both at 10 µg/ml) was added to polarized cells from apical and basolateral chambers. Cells were grown for 3 days, and culture medium was changed daily to add fresh antibodies. Viral plagues were quantitated as described above.

### Western Blot Assay

For Western blot detection of E-cadherin and nectin-1, polarized tonsil epithelial cells were extracted, and proteins were separated on a 16% SDS-polyacrylamide gel and blotted with anti-E-cadherin and nectin-1 antibodies (both from Invitrogen/Life Technologies). The protein bands were visualized using ECL detection reagents (GE Healthcare Bio-Sciences, Pittsburgh, PA), and equal protein loading was confirmed by detection of β-actin (Ambion/Life Technologies).

### Detection of Nectin-1 and HSV-1 gD on the Cell Surface by Domain-Selective Labeling Assay

Polarized cells were labeled with 200 µg/ml sulfo-NHS-LC-biotin (Thermo Fisher Scientific Inc., Rockford, IL) from apical or basolateral membranes for 30 min as described [44], [47]. Cells were washed with Tris saline (10 mM Tris-HCl pH 7.4, 120 mM NaCl), and extracted in lysis buffer containing 1% Nonidet P-40, 1% sodium deoxycholate, 0.1% sodium dodecyl sulfate (SDS), 1 mM phenylmethylsulfonyl fluoride (PMSF), and aprotinin (1 µg/ml). Biotinylated proteins were precipitated with streptavidin–agarose beads in lysis buffer (Thermo Fisher Scientific, Waltham, MA), followed by separation on a 4–20% Tris-glycine SDS-polyacrylamide gel and transferred to nitrocellulose membranes (GE Healthcare, Germany). Nectin-1 was detected using mouse monoclonal (Invitrogen/Life Technologies) antibodies. To determine binding of HSV-1 gD to nectin-1, we used the truncated form of HSV-1 gD ectodomain gD(306t). HSV-1 gD(306t) at 20 µg/ml was added to apical or basolateral membranes for 30 min, and cells were washed and subjected to domain-selective labeling from appropriate cell membranes. HSV-1 gD was detected using rabbit polyclonal antibodies (MyBioSource). Bands were visualized using the ECL detection system (GE Healthcare).

### MAPK Activation Assay

Polarized tonsil epithelial cells were incubated with HIV tat and/or gp120 recombinant proteins and UV-inactivated or active cell-free HIV virions. One set of cells was treated with MAPK inhibitor U0126 (Sigma) at 1 µM. The absence of toxic effect of 1 µM U0126 on polarized cells was confirmed by MTT cell viability assay (Biotium Inc.) and measurement of TER. After 5 days, cells were extracted and proteins were separated on a 4–20% Tris-glycine SDS-polyacrylamide gel and transferred to nitrocellulose membranes (GE Healthcare). MAPK signaling activity was measured by detection of phosphorylated and total ERK1/2 protein using mouse monoclonal antibodies (Cell Signaling Technology, Danvers, MA) that recognize phosphorylated (Clone E10, #9106) and nonphosphorylated (#9102) ERK1/2. Protein bands were visualized using the ECL detection system (GE Healthcare). Inhibition of MAPK activity by U0126 was quantitated by measuring the intensity of pixels (mean density) in protein bands using Image J software.

### Statistical Analysis

Data are presented as mean ± standard error of the mean (SEM) of two experiments performed in duplicate. The differences between control cells (untreated or treated with inactive tat/gp120) and experimental cells (treated with active tat/gp120 and HIV virions) were analyzed using Student's *t* test. A p value of <0.05 was considered significant.

## Results

### HIV Tat- and gp120-Induced Disruption of Tight Junctions of Polarized Oral Epithelial Cells Facilitates Paracellular Spread of HSV-1

**Figure 1:**
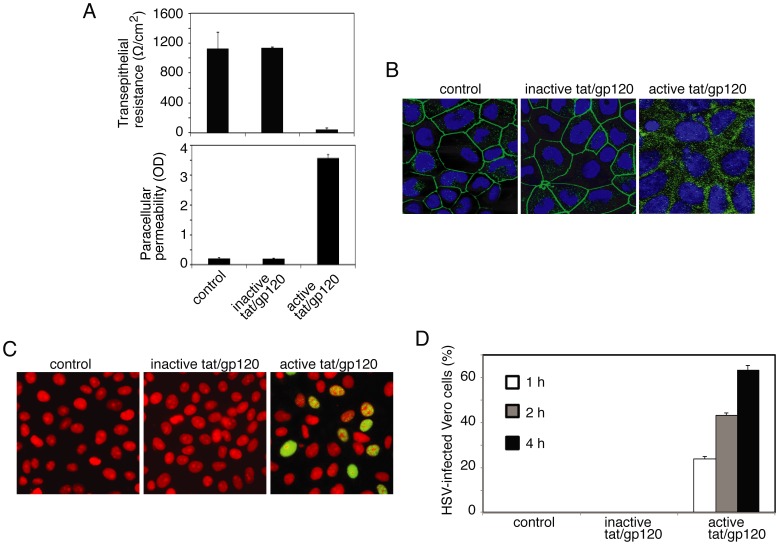
HIV tat- and gp120-induced disruption of TJs of oral epithelial cells facilitates the paracellular spread of HSV-1. **A** (upper panel). Polarized tonsil epithelial cells were treated with active or inactive recombinant HIV tat and gp120 in combination for 5 days, and TER was then measured. **A** (lower panel). The same cells were used to evaluate paracellular permeability after 5 days of treatment, as determined by leakage of IgG (Fab’)_2_ from the apical chamber to the basolateral chamber. OD, optical density. **B.** The same cells were immunostained for ZO-1 (green). Cell nuclei are stained in blue. **C.** HSV-1 at an MOI of 10 PFU per cell was added to the upper chamber of polarized cells, and culture medium was collected from the basolateral chamber after 1, 2, or 4 h. HSV-1 paracellular spread was confirmed by detection of ICP4 protein in Vero cells (green) 4 h after infection. Cell nuclei were stained with propidium iodide (red). Yellow indicates colocalization of ICP4 with the nuclear marker. **D.** HSV-1 paracellular spread was quantified by counting of HSV-1-infected Vero cells in 10 random microscopic fields and determining the percentage of cells positive for ICP4. **A,** **D:** Error bars indicate SEM (n = 3).

The tight junctions of oropharyngeal mucosal epithelium have critical barrier functions for protection against paracellular spread of various pathogens, including viruses. We have shown that HIV-associated disruption of tight junctions of oral mucosal epithelium facilitates paracellular spread of human papillomavirus [15]. HIV tat and gp120 had the greatest effect on TJ disruption when cells were treated with a combination of the two proteins for 5 days, and there was no toxic effect on the cells [15]. Thus, to determine if HIV tat- and gp120-induced TJ disruption facilitates HSV-1 paracellular spread, we treated polarized tonsil epithelial cells with a combination of recombinant tat and gp120 at 10 ng/ml each, which is similar to physiological levels in HIV-infected individuals [54], [55], [56], [57] (Fig. 1A). As negative controls we used mutant tat protein, which lacks the basic and integrin-binding domains, and heat-inactivated gp120; both are functionally inactive proteins [15], [21], [58], [59], . Culture medium was changed daily to include fresh proteins, and disruption of TJs was confirmed on day 5 in two inserts of each plate by three independent assays in the following order: (i) measurement of TER, (ii) evaluation of paracellular permeability, and (iii) immunostaining of cells for TJ protein ZO-1. Measurement of TER showed that the electrical resistance of cells treated with active forms of tat and gp120 was substantially reduced, in contrast to that of TER in control cells, either untreated or treated with inactive tat and gp120 (Fig. 1A, upper panel). Consistent with the TER data, paracellular leakage of IgG (Fab’)_2_ from the apical chamber to the basolateral chamber was detected only when cells were treated with active tat/gp120 (Fig. 1A, lower panel). Finally, localization of ZO-1 in polarized cells treated with active tat/gp120 was diffuse cytoplasmic, indicating disruption of TJs (Fig. 1B). Polarized cells treated with inactive tat and gp120 and untreated control cells showed the localization of ZO-1 as a ring shape, which is typical for intact TJs (Fig. 1B).

After confirmation of TJ disruption, we added HSV-1 to the apical surface of polarized cells at an MOI of 10 PFU per cell for 1, 2, or 4 h. Measurement of TER in control cells exposed to HSV-1 showed that TER was not reduced at any time point (data not shown), indicating that HSV does not alter the TJs of tonsil epithelial cells. At various times, the culture medium in the lower chamber was collected for the HSV-1 infectivity assay in Vero cells. Virus-containing basolateral culture medium was added to Vero cells, and culture was maintained for 4 h. Vero cells were then fixed and immunostained for HSV-1 ICP-4, which is immediate-early protein expressed at 4 h after infection (Fig. 1C) [61]. Confocal immunofluorescence analysis of Vero cells showed that ICP4 was expressed in the nuclei of cells treated with active tat/gp120 and exposed to HSV-1. In contrast, ICP4 was not detected in Vero cells that were untreated or treated with inactive tat/gp120 and exposed to HSV-1. Quantitative analysis of HSV-infected Vero cells indicated that HSV paracellular spread through tat/gp120-disrupted TJs occurred after 1 h of exposure to HSV-1 from the apical surface (Fig. 1D). During the next 2 and 4 h of HSV-1 exposure, the paracellular spread of HSV increased in a time-dependent fashion. These data indicate that HIV tat/gp120-induced disruption of TJs of tonsil epithelial cells facilitates the paracellular spread of HSV-1.

To determine if interaction of HSV-1 with oral epithelial cells disrupts epithelial TJs, polarized tonsil epithelial cells were treated with one of the key glycoproteins of HSV-1, the soluble gD(306t), which contains 1–306 residues in its extracellular domain. Cells were treated with 10 ng/ml of gD for 5 days and culture media with fresh protein was changed every day. Measurement of TER and immunostaining of ZO-1 showed that HSV-1 gD did not reduce TER and did not alter localization of ZO-1 of polarized cells (data not shown), indicating that HSV gD is not involved in disruption of TJs.

### Interaction of HIV Virions with Mucosal Epithelium Disrupts Epithelial Tight Junctions and Promotes HSV Paracellular Spread

**Figure 2:**
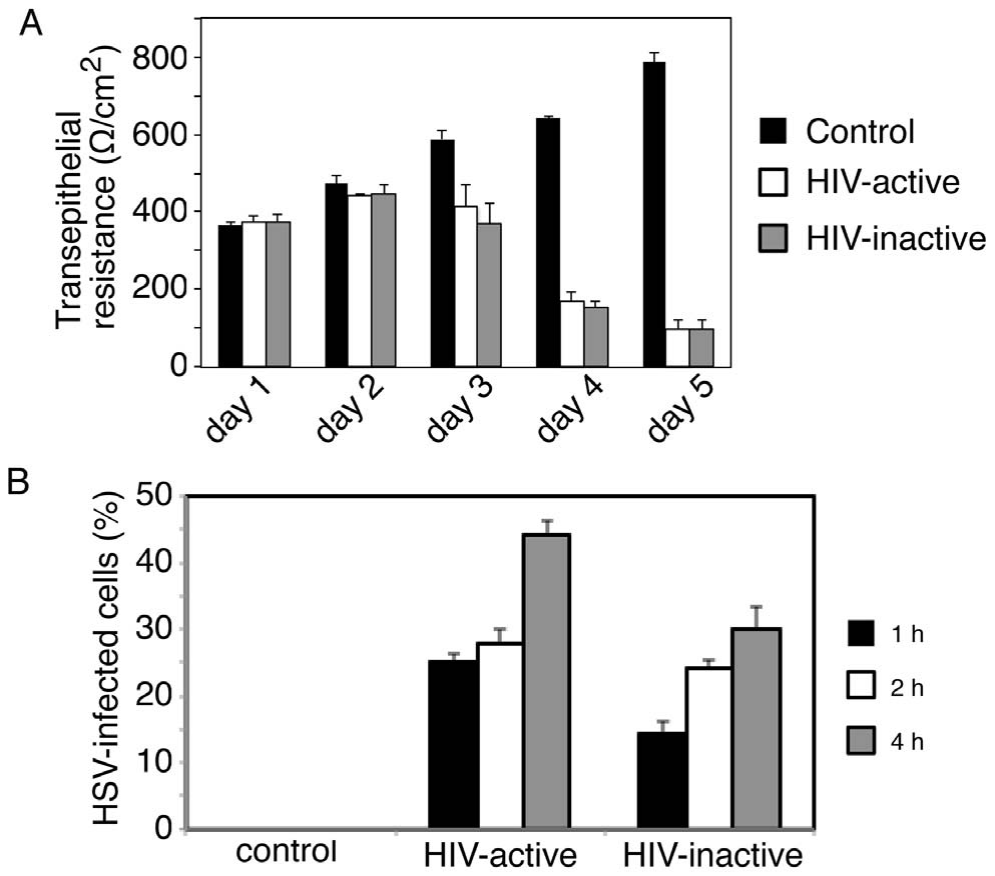
HIV cell-free virion-associated disruption of epithelial tight junctions facilitates HSV paracellular spread. **A.** Polarized tonsil epithelial cells were incubated with dual X4- and R5-tropic HIV-1_SF33_ for 5 days. One set of cells was exposed to UV-inactivated virions. Culture medium was changed daily to add fresh virus, and TER was measured. **B.** HSV-1 was added to the apical surface of polarized cells upon complete disruption of TJs at 5 days. HSV paracellular spread at 1, 2, and 4 h after incubation was examined in Vero cells grown in the basolateral chamber of filter inserts by immunostaining of ICP4 protein. HSV-1 paracellular spread was quantified by counting HSV-1-infected Vero cells, and the percentage of cells positive for ICP4 was determined. **A, B:** Error bars indicate SEM (n = 3).

To determine whether direct interaction of cell-free HIV virions with mucosal epithelium disrupts the TJs, we exposed polarized tonsil epithelial cells to dual X4- and R5-tropic HIV-1_SF33_ for 5 days. In parallel experiments UV-inactivated virions were used. Culture medium was changed daily to add fresh virus, and TER was measured daily. In untreated control cells, TER increased over the 5 days. In contrast, TER gradually declined in cells exposed to either inactivated or active HIV virions (Fig. 2A). The HIV-induced reduction in TER was detected only with prolonged exposure to virus (4–5 days); with shorter treatment (1–3 days) it was not. Analysis of paracellular leakage of IgG (Fab’)_2_ confirmed the TER data; i.e., both inactive and active HIV disrupted epithelial TJs (data not shown). To examine the paracellular spread of HSV through HIV-disrupted tonsil epithelial cells, we added HSV to the apical membranes of polarized cells for 1, 2, or 4 h and tested the basolateral medium for HSV infection in Vero cells. Quantitative analysis of HSV-infected Vero cells showed HSV paracellular spread through cells disrupted by both HIV-inactivated and HIV-active virus (Fig. 2B). HSV paracellular spread was not detected through control cells with intact TJs.

### HIV tat and gp120 and Cell-free HIV Virions Induce Activation of MAPK Signaling in Polarized Oral Epithelial Cells

**Figure 3:**
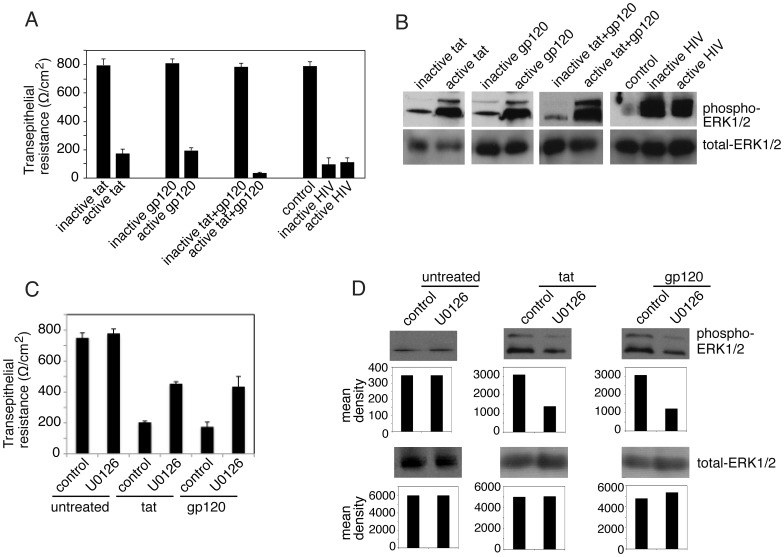
HIV tat and gp120 activate MAPK in polarized oral epithelial cells. (**A**) Polarized cells were treated with active or inactive tat and gp120, alone or in combination. In parallel experiments, cells were exposed to UV-inactivated or active HIV-1_SF33_. Culture medium was changed daily to add fresh proteins and virus, and at day 5 the TER was measured. (**B**) After measurement of TER, the same cells were used for evaluation of MAPK activation. Cells were extracted, and total and phosphorylated ERK1/2 were detected by Western blot assay. (**C**) Polarized cells were treated with active forms of tat or gp120 in the presence or absence of MAPK inhibitor U0126. Tat -and gp120 -untreated cells with or without U0126 served as controls. At day 5 TER of polarized cells was measured. (**D**) The same cells from panel C after measurement of TER were extracted and used for evaluation of phosphorylated and total ERK1/2. The mean densities of pixels in the protein bands were measured by Image J software, and the results for each gel are shown as a bar graph under each blot. **A and C:** Error bars indicate SEM (n = 3).

MAPK activation is a key mechanism for the disruption of TJs, and HIV tat binds to β1 and αϖ integrins [59], [60], [62], [63], which leads to induction of MAPK activation in endothelial cells [21], [22], [23]. Binding of HIV gp120 to chemokine receptors CXCR4 and CCR5 in lymphocytes and macrophages also leads to induction of MAPK [64], [65], [66], [67]. We have shown that polarized oral epithelial cells express to β1 and ϖ integrins, and CCR5 and CXCR4 [44], [46], [68], indicating availability of HIV tat and gp120 receptors in oral epithelium. To determine if HIV tat and gp120 interaction with these receptors induces MAPK in oral epithelial cells, we examined the phosphorylation of MAPKs ERK1/2 in polarized tonsil epithelial cells. Cells were treated with tat or gp120 alone or in combination for 5 days. Also, tonsil cells were incubated with UV-inactivated and infectious cell-free HIV virions. TER was drastically reduced (80–90%) in cells treated with active tat and/or gp120 and with active or inactive HIV virions, compared to the TER of untreated control cells and cells treated with inactive tat and/or gp120 (Fig. 3A). After confirmation of TJ disruption, cells were extracted and examined for total and phosphorylated ERK1/2 by Western blot assay (Fig. 3B). These results show that both active tat and gp120 induce ERK1/2 phosphorylation and that treatment of cells with a combination of tat and gp120 substantially increases ERK1/2 phosphorylation over that in cells treated with tat or gp120 alone. Analysis of MAPK activation in the cells incubated with UV-inactivated and infectious HIV virions showed that both inactive and active virions induce phosphorylation of ERK1/2.

To confirm the role of MAPK signaling in HIV-associated disruption of TJs, polarized cells were treated with tat or gp120 in the presence or absence of MAPK inhibitor U0126 at 1 µM (Fig. 3C). Culture media with fresh proteins and inhibitor were changed every day, and at day 5 TER was measured. Comparison of TER in tat or gp120-treated cells with and without U0126 showed that TER was increased about 2 fold in cells with U0126 compared to cells without MAPK inhibitor (Fig. 3C). In the presence of U0126, TER reached almost 60% of its normal level in tat or gp120-untreated cells. The presence of U0126 in tat- or gp120-untreated cells did not alter the TER. Analysis of ERK1/2 phosphorylation in tat- or gp120-treated cells in the presence and absence of U0126 showed that U0126 induced approximately 50% reduction of ERK1/2 phosphorylation, which was well correlated with the increase of TER (Fig. 3D). These findings indicate that inhibition of MAPK signaling prevented tat and gp120-induced disruption of tight junctions, i.e., HIV-induced MAPK activation is critical for disruption of TJs in oral epithelial cells.

### HIV tat and gp120 and Cell-free HIV Virions Disrupt Adherens Junctions of Oral Epithelial Cells and Expose Nectin-1

Activation of MAPK signaling may also lead to disruption of AJs [69], [70]. Dissociation of AJs by calcium depletion liberates nectin-1, facilitating HSV-1 infection [43]. We hypothesized that HIV-induced MAPK activation may also disrupt AJs, liberate nectin-1, and thereby promote HSV-1 infection. To test this hypothesis, we investigated the role of HIV tat/gp120 and cell-free HIV virions in the disruption of AJs, liberation of nectin-1, and binding of nectin-1 to HSV gD.

**Figure 4:**
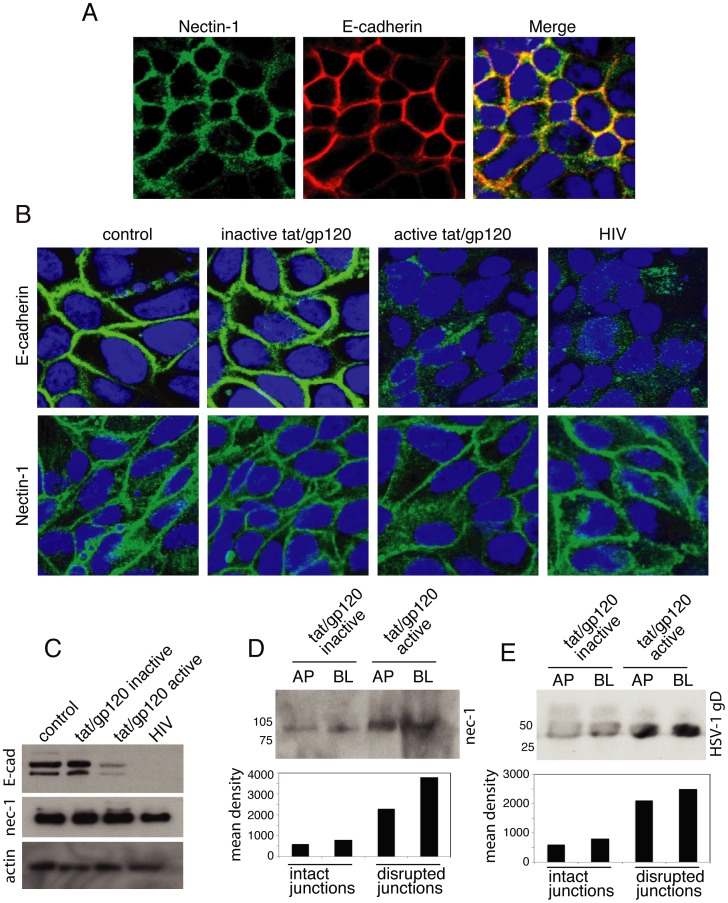
HIV-disrupted epithelial junctions lead to exposure of nectin-1 and facilitate its binding to HSV-1 gD. **A.** Polarized tonsil epithelial cells were coimmunostained for nectin-1 and E-cadherin. Yellow in the merged panel indicates colocalization of nectin-1 and E-cadherin. **B.** Polarized tonsil cells were treated with active or inactive tat and gp120 in combination for 5 days. In parallel experiments, cells were exposed to HIV-1_SF33_ for 5 days. Cells were then immunostained for E-cadherin and nectin-1. **C.** Polarized cells were treated with active or inactive tat and gp120 in combination or with cell-free HIV-1_SF33_ for 5 days. Cells were then extracted, and E-cadherin and nectin-1 were detected by Western blot assay. **D.** Apical or basolateral membranes of polarized epithelial cells were treated with inactive or active HIV tat and labeled with sulfo-NHS-LC-biotin. Nectin-1 was detected in the avidin-precipitated total membrane proteins by Western blot assay. **E.** HSV-1 gD(306t) at 20 µg/ml was added to apical or basolateral membranes of polarized epithelial cells treated with inactive or active HIV tat/gp120. After 30 min the cell surface was labeled with sulfo-NHS-LC-biotin. Proteins biotinylated at the cell surface were precipitated with streptavidin–agarose beads, and gD was detected by Western blot assay. AP, apical; BL, basolateral.

Coimmunostaining of nectin-1 and E-cadherin in polarized tonsil epithelial cells showed that these two adhesion proteins were colocalized (Fig. 4A). To examine whether the HIV tat and gp120 and virions disrupt the AJs, we treated polarized tonsil cells with inactive or active tat and gp120 in combination and cell-free HIV virions for 5 days. Immunostaining of E-cadherin in these cells showed that active tat/gp120 as well as cell-free HIV virions substantially reduced or completely inhibited expression of E-cadherin and altered its localization from cell membrane to cytoplasm (Fig. 4B). In contrast, E-cadherin in cells treated with inactive tat and gp120 and in untreated control cells was exclusively localized in the cell membranes, where AJs are formed. Nectin-1 expression by active tat/gp120 and HIV virions was not reduced, and its localization was not changed (Fig. 4B). A Western blot assay confirmed the immunostaining data (Fig. 4C). Treatment of polarized cells with HSV-1 envelope proteins gD(306t) for 5 days did not alter localization of E-cadherin and nectin-1 (data not shown), indicating that HSV-1 gD does not disrupt AJs.

To determine if disruption of AJs liberates nectin-1, we performed domain-specific surface labeling of apical or basolateral membranes of polarized cells with sulfo-NHS-LC-biotin. The presence of nectin-1 was examined in the avidin-precipitated total membrane proteins by Western blot assay. Analysis of nectin-1 in the apical and basolateral membranes of polarized cells treated with inactive tat and gp120 showed that only a trace amount of nectin-1 was present in the apical and basolateral membranes of cells with an intact AJ (Fig. 4D). In contrast, substantially more nectin-1 was detected on the polarized cells with AJs disrupted by active tat and gp120. These data indicate that the disruption of tat/gp120-induced AJs allows for more penetration of biotin into junctional areas, where it subsequently labeled nectin-1. Penetration of biotin into nectin-1 sequestered areas without relocalization of nectin-1 from the lateral membranes showed that disruption of AJs did not lead to liberation or release of nectin-1. More likely, disruption of AJs facilitated exposure of nectin-1 to cell surface biotinylation; i.e., sequestered nectin-1 within the AJs was exposed owing to the disruption of AJs.

To study the role of HIV tat/gp120-exposed nectin-1 in HSV-1 gD binding, we performed soluble gD binding to apical and basolateral surfaces of intact and disrupted polarized cells by active and inactive tat/gp120, respectively. The soluble HSV-1 gD(306t), which binds to nectin-1, was added to apical or basolateral membranes of polarized cells for 30 min. The apical or basolateral surfaces of polarized cells were biotinylated, and the presence of HSV gD was examined in the avidin-precipitated total membrane proteins by Western blot assay. Domain-specific labeling showed that HSV-1 gD binding to the cell surface was increased two- to threefold in the polarized cells with the disrupted AJs than in the cells with intact AJs (Fig. 4E), indicating that the exposure of nectin-1 is facilitated by HSV-1 gD binding to disrupted epithelial cells.

### HIV-Induced Exposure of Nectin-1 Facilitates HSV-1 Infection

**Figure 5:**
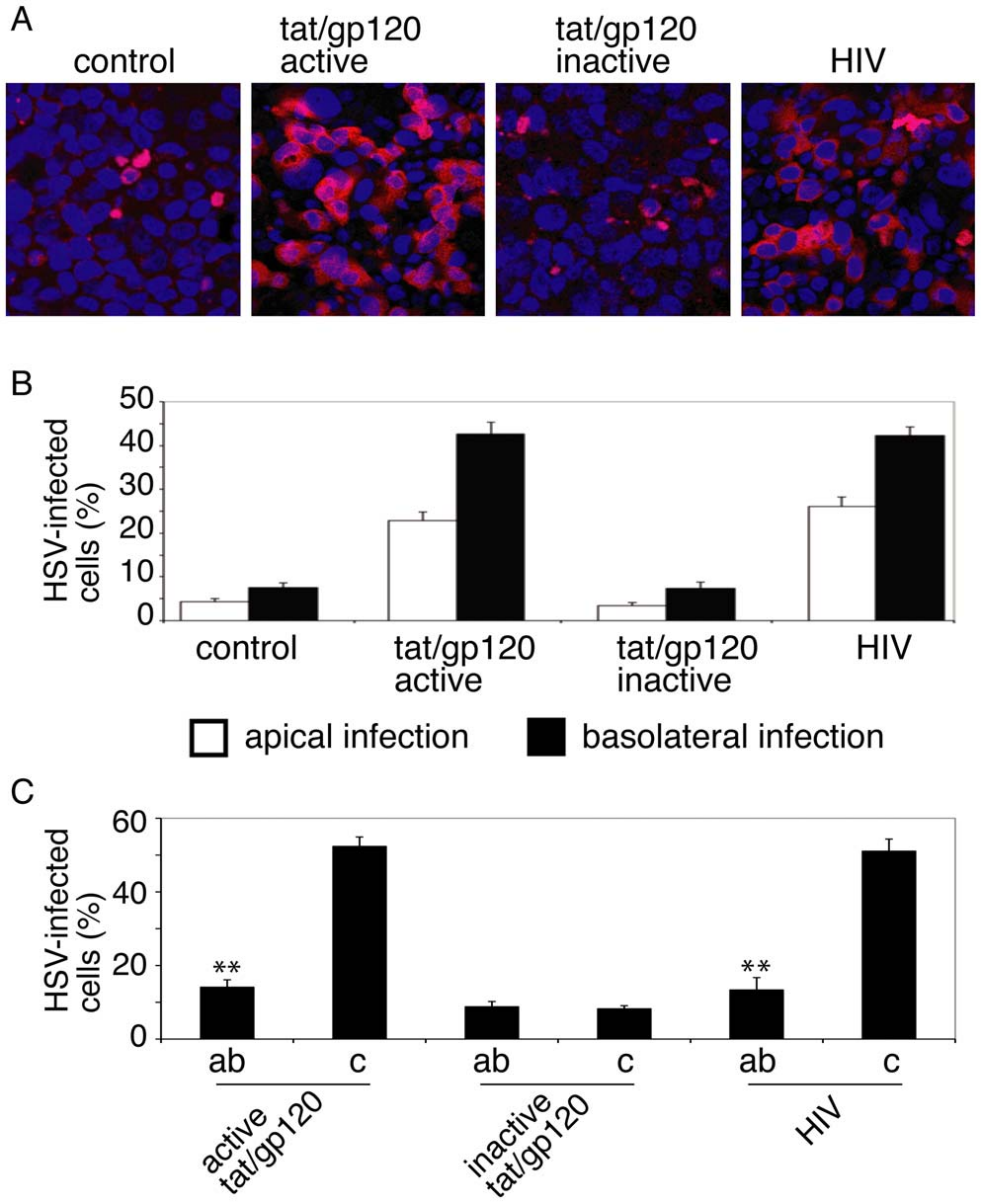
HIV-disrupted epithelial junctions facilitate HSV-1 infection. **A.** Polarized tonsil cells were treated with HIV tat/gp120 or HIV virions for 5 days and infected with HSV-1. After 24 h, cells were fixed and immunostained using anti-HSV-1 gB antibodies (red). Cell nuclei are stained in blue. **B.** HSV-1 infection was quantitatively evaluated, and the percentage of cells positive for gB was determined. Error bars indicate SEM. **C.** Cells were incubated with antibodies against nectin-1 for 1 h and then infected with HSV-1. Cells were fixed after 24 h, HSV-1 infection was confirmed by detection of goat anti-HSV-1 immune serum, and the number of infected cells was counted. ab, cells incubated with antibodies. c, control cells without antibodies. Error bars indicate SEM. *P<0.01, **P<0.001, all compared with the control group.

To determine if HIV-induced exposure of nectin-1 plays a role in HSV infection, we examined HSV-1 infection in polarized cells with intact AJs or AJs disrupted by active or inactive tat/gp120. HSV infection was also examined in cells with AJs disrupted by HIV virions. Infected cells from apical or basolateral surfaces were evaluated by immunostaining of cells with monoclonal antibody against HSV-1 gB. Confocal microscopic analysis showed that most HSV-1 gB-positive cells were found in the cells with AJs disrupted by active tat/gp120 or HIV virions (Fig. 5A). Quantitative analysis showed that HSV infection was four- to fivefold higher in the cells with disrupted AJs than in the cells with intact AJs (Fig. 5B). HSV infection from the apical surface was approximately one-half that from the basolateral surface (Fig. 5B).

To further investigate the role of nectin-1 in HSV entry into polarized cells with disrupted AJs, we treated polarized tonsil cells with active or inactive tat/gp120 and HIV virions for 5 days. After confirmation of AJ disruption, cells were preincubated with antibodies to nectin-1 (CK-8), which recognizes its V domain between 80 and 105 aa where it binds HSV-1 gD [71]. Cells were then infected with HSV-1. Cells without antibodies served as a control. After 24 h, cells were fixed and immunostained with goat anti-serum against HSV-1. Quantitative analysis of HSV-infected cells showed that antibodies to nectin-1 reduced HSV-1 infection in cells treated with active tat/gp120 and HIV virions by ∼70% (Fig. 5C). Significant inhibition of HSV-1 infection by anti-nectin-1 antibodies in cells treated with inactive tat/gp120 was not observed, indicating that antibodies are efficient when AJs are disrupted. These data clearly indicate that tat/gp120- and virion-induced disruption of AJs exposes nectin-1 to gD, which binds to nectin-1, facilitating viral infection. However, approximately 10–15% of cells with intact junctions were infected with HSV-1 in the presence of anti-nectin-1 antibodies. This could be basal HSV-1 infection of polarized cells due to low levels of other gD receptors, including herpesvirus entry mediator (HVEM) and 3-O-sulfated heparan sulfate (3-OS-HS), which are not restricted to junctions [72], [73], [74], [75].

### HIV-Induced Exposure of Nectin-1 Facilitates HSV-1 Cell-to-Cell Spread

**Figure 6:**
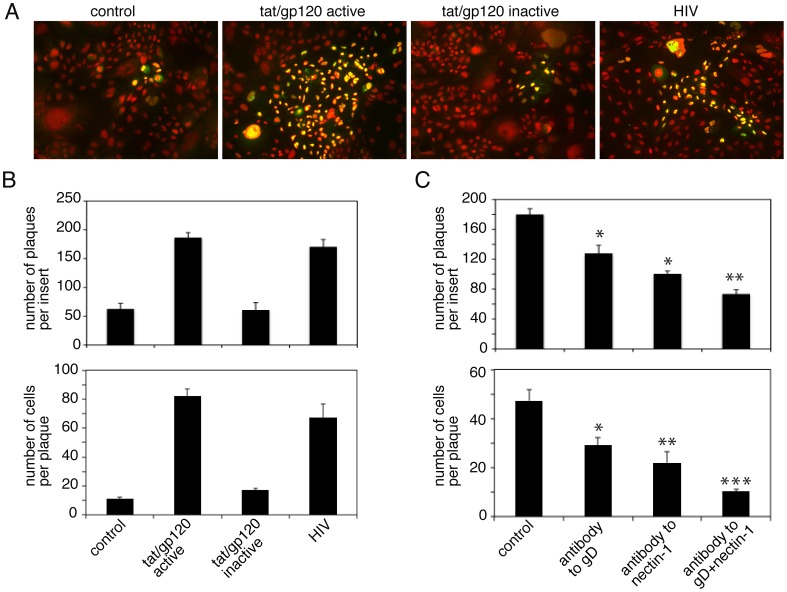
HIV tat/gp120-disrupted epithelial junctions facilitate HSV-1 cell-to-cell spread through junctional areas of polarized oral epithelial cells. **A.** Polarized tonsil cells were treated with active or inactive tat/gp120 and HIV virions for 5 days. Disrupted cells were infected with HSV-1 at 0.01 PFU per cell from basolateral membranes of polarized cells. After 3 days, cells were fixed and immunostained using goat anti-HSV immune serum (green). Cell nuclei are stained in red. Yellow represents colocalization of HSV proteins and nuclei. **B.** (upper panel) Plaque numbers were counted from 3 independent filter inserts and data are presented as the average number of HSV-infected plaques per insert. (lower panel) Cell-to-cell spread of HSV-1 was quantitatively evaluated by counting HSV-infected cells in the plaques. Results are presented as the average number of HSV-infected cells per plaque. Error bars indicate SEM. **C.** Polarized cells were infected with HSV-1. After 4 h, antibodies to nectin-1 and gD were added separately and in combination. Cell medium was changed daily to add fresh antibodies. Cells were fixed and immunostained for HSV-1, and the plaque numbers (upper panel) and the number of HSV-1-positive cells in plaques were counted (lower panel). Error bars indicate SEM. *P<0.05, *P<0.01, **P<0.001, all compared with the control group.

Since interaction of HSV-1 gD with nectin-1 is critical for cell-to-cell spread of virus [35], [76], [77], [78] we investigated the role of HIV-associated exposure of nectin-1 in the spread of HSV-1. Polarized tonsil cells were treated with active or inactive tat/gp120 and HIV virions, and disruption of epithelial junctions was confirmed by measurement of TER after 5 days. Cells were infected with HSV-1 from basolateral membranes at an MOI of 0.01 PFU per cell and were fixed and immunostained with polyclonal goat anti-HSV serum after 3 days. Confocal microscopy of cell-to-cell spread of virus revealed small foci (plaques) of infected cells in the untreated control cells and in the cells treated with inactive tat/gp120 (Fig. 6A). HSV-infected plaques in the cells treated with active tat/gp120 and HIV virions were substantially larger. Analysis of HSV-infected plaque numbers also showed that about 4 fold more plaques were detected in disrupted cells by tat/gp120 and HIV virions than in intact cells of untreated controls and cells treated with inactive tat/gp120 (Fig. 6B, upper panel). Quantitative analysis of plaque size showed that the average number of infected cells in untreated control cells and in cells treated with inactive tat/gp120 was approximately 10–15 cells per plaque (Fig. 6B, lower panel). In contrast, the number of infected cells in polarized cells treated with active tat/gp120 and HIV virions was 70–80 cells per plaque.

Next, we incubated cells with active tat/gp120 for 5 days. After confirming the reduction of TER, we exposed cells to HSV-1; after 2 h, the infected cells were incubated with antibodies against gD, nectin-1, and a combination of gD and nectin-1. Cells were maintained for 3 days, and culture medium was changed every day to add fresh antibodies. Quantitative analysis of HSV-infected plaque numbers showed that anti-gD and -nectin-1 antibodies independently reduced plaque numbers by approximately 30% and 40%, respectively, compared to control cells without antibodies (Fig. 6C, upper panel). Combination of these two antibodies reduced plaque numbers by about 60%. Quantitative analysis of plaque formation showed that antibodies to gD and nectin-1 independently reduced plaque size by approximately 60% and 50%, respectively (Fig. 6C, lower panel). Furthermore, the combination of antibodies to nectin-1 and gD reduced plaque size by about 70%. These data indicate that HIV-induced disruption of AJs and exposure of nectin-1 are important for the cell-to cell spread of HSV in oral epithelial cells.

## Discussion

We have shown that HIV proteins tat and gp120 and cell-free HIV virions facilitate HSV infection and spread within the oral epithelium. HIV tat-, gp120-, and virion-induced disruption of TJs and AJs of oral epithelial cells facilitates HSV-1 paracellular spread. Furthermore, HIV tat/gp120- and HIV virion-induced disruption of AJs of oral epithelial cells substantially increases HSV infection and cell-to-cell spread.

The barrier function of the lining of oropharyngeal, gut, and anogenital mucosal epithelium is mediated by well-developed epithelial TJs and AJs. HIV-associated disruption of TJs opens paracellular space between epithelial cells, leading to the passage of multiple pathogens, including viruses [15], [19], [50], [79]. The loss of barrier function in mucosal epithelium may promote reinfection by HSV-1 with the same or different genotype; this is possible in HSV-seropositive individuals [80], [81], [82], [83], [84]. Moreover, HIV-induced dysfunction of the immune system may increase reinfection by HSV with different genotypes of virus [85]. The opening of paracellular space between mucosal epithelial cells may also facilitate the paracellular release of reactivated HSV into saliva, leading to the rapid spread of virus to others.

HIV-induced disruption of both TJs and AJs depends on MAPK activation by tat and gp120 and HIV virions. It is well documented that the MAPK-associated mechanism of HIV tat- and gp120-induced disruption of TJs takes place through aberrant internalization of TJ proteins and their down-regulation and/or proteasome-mediated degradation [21], [22], [23], [24], [25], [26], [27], [28], [29], [30], [31], [32]. Here we have shown for the first time that HIV tat and gp120 proteins and HIV virions also induce dissociation of AJs. It has been reported that activation of the MAPK ERK pathway in epithelial cells induces expression of transforming growth factor-β1, ωηιχη causes internalization of E-cadherin from AJs into cytoplasm, λεαδing to the epithelial-to-mesenchymal transition (EMT) [86]. MAPK activation also induces the expression of fibroblast growth factor-2, which causes E-cadherin down-regulation via EMT mechanisms [87]. Our data show that HIV virions and tat/gp120 induce activation of MAPK ERK1/2 and reduce E-cadherin expression, also altering its localization from membrane to cytoplasm. These findings suggest that HIV induces the disruption of AJs through the EMT phenotype, which is a well-coordinated epigenetic process [88]. HIV has previously been shown to cause EMT in renal epithelium. HIV infection is associated with kidney failure due to severe nephropathy, characterized by the loss of the renal epithelial phenotype and the acquisition of mesenchymal features, including de-differentiation, depolarization, and proliferation [89], [90], [91], [92], [93]. Our data also consistently show that treatment of polarized oral epithelial cells with HIV tat and gp120 and HIV virions induces disruption of TJs and AJs and therefore the depolarization of epithelial cells. UV irradiation of HIV virions did not affect the role of HIV in the disruption of cell junctions and depolarization, indicating that HIV infection is not critical for disruption of cell junctions.

The major receptor for HSV-1 gD nectin-1 is an adhesion protein associated with AJs. Nectin-1 has hemophilic interactions with itself and heterophilic interactions with nectins 3 and 4; such interactions within the lateral membranes of epithelial cells form AJs [94]. Dissociation of AJs by calcium depletion liberates nectin-1, indicating that it is sequestered within the AJs [43]. However, in our study HIV tat/gp120- and HIV-induced disruption of AJs did not alter localization of nectin-1 from lateral membranes of polarized cells, suggesting that, in this case, release or liberation of nectin-1 may not occur. Rather, our results show that HIV-associated disruption of AJs leads to the exposure or unmasking of nectin-1 from its sequestered areas. HIV-induced exposure of nectin-1 is a key factor for HSV infection in the lateral membranes of oral epithelial cells. Nectin-1 has three Ig-like extracellular domains; HSV gD binds to V domain, which is the distal Ig-like domain [95], [96]. Antibodies to the V domain of nectin-1 and to HSV gD reduce HSV-1 infection, indicating that HIV-induced disruption of AJs exposes the V domain of nectin-1 to the HSV-1 gD. This notion is well supported by the lack of inhibitory effect of anti-nectin-1 antibodies to HSV-1 infection in cells with intact AJs; i.e., nectin-1 is hidden within the intact AJs and thus is not accessible to the HSV gD. HSV-1 entry may occur by direct fusion of the viral envelope with the plasma membrane or by endocytosis of virions into the cytoplasm and subsequent fusion of the virion envelope with endosomal membranes [97], [98]. Mechanisms of HSV gD and –nectin-1-mediated viral entry via HIV-disrupted junctional areas need to be clarified.

HIV-induced disruption of AJs also promotes the cell-to-cell spread of HSV-1, indicating that the availability of the nectin-1 V domain on the lateral membranes of epithelial cells is critical for the spread of progeny virions. It has been well documented that the extracellular V domain of nectin-1 is critical for HSV cell-to-cell spread, in contrast to the cytoplasmic tail and transmembrane domains of nectin-1, which do not play a critical role in virus spread [76]. HSV-1 does not spread from nectin-1-positive to nectin-1-negative cells [77], suggesting that the disruption of AJs may efficiently promote virus spread as it exposes nectin-1 from neighboring epithelial cells. During the spread of HSV, newly synthesized gD replaces the nectin-1 in infected cells at junctions with uninfected cells [78]. This requires that unoccupied nectin-1 binding sites be available from hemophilic or heterophilic interactions of nectin-1 from uninfected cells. Thus, the HIV-induced exposure of nectin-1 promotes binding of gD to unoccupied nectin-1 binding sites from uninfected cells and therefore may facilitate rapid HSV cell-to-cell spread.

HSV gD also interacts with other receptors. HSV-1/2 gD binds to HVEM, HSV-1 gD binds to 3-OS-HS, and HSV-2 gD interacts with nectin-2 [72], [73], [74], [75], [99]. Further study may elucidate the functional role of these receptors in HSV infection and cell-to-cell spread in HIV-disrupted polarized oral epithelial cells.

HIV-associated HSV-1 spread in the oral epithelium is a common manifestation of HIV/AIDS [2], [3], [4], [5], [6], [7], in which junctions of oral epithelium are disrupted [15]. HIV-associated exposure of nectin-1 in oral epithelium could be an important mechanism for the rapid spread of HSV-1 infection. Furthermore, our findings may also apply to HIV-associated spread of HSV-2 within the genital mucosa [100], [101], [102], [103], [104], because HIV induces disruption of vaginal and cervical epithelium [50] and HSV-2 also uses nectin-1 for entry and spread [105].

The presence of HIV tat and gp120 in the mucosal environment of HIV-infected individuals is critical for the disruption of epithelial junctions and the exposure of nectin-1. The presence of HIV virions and HIV gene products in oral and genital epithelium is well described [15], [106], [107], [108], [109], [110], [111], [112], [113], [114], [115], [116], [117], [118], [119], especially in circulating HIV-infected immune cells [117], [118], [119]. Secretion of HIV tat and gp120 into blood has been shown [54], [55], [56], [57], [120], [121], [122]. The immune cells expressing tat and gp120 have been detected in the oral and anal mucosa of HIV-infected individuals, including those on antiretroviral therapy (ART) [15]. HIV in anal tissues, including tissues from ART-treated donors, replicates and is infectious [15]. Furthermore, tat and gp120 are detected in the saliva, and salivary tat penetrates mucosal epithelium [15]. HIV tat may penetrate cells and tissues through its protein transduction domain, which is based on the amino acids arginine and lysine. Internalization of protein into cells and tissues is accomplished by several mechanisms, including endocytosis and macropinocytosis [123], [124], [125], [126], [127]. Mucosal epithelium may therefore be exposed to tat and gp120 from multiple sources, including saliva and circulating immune cells, even among individuals receiving ART whose HIV viral load is suppressed. Mucosal epithelium may also serve as an HIV reservoir [118] and as a source of tat and gp120, which disrupt AJs and expose nectin-1, leading to infection and spread of HSV-1 and -2.

Incubation of polarized oral epithelial cells with cell-free HIV virions also caused disruption of TJs and AJs and exposed nectin-1, leading to HSV-1 infection and spread. Thus, in addition to secreted tat and gp120, the presence of HIV virions within the mucosal environment may contribute to the disruption of mucosal epithelium and thereby the promotion of HSV spread.

**Figure 7:**
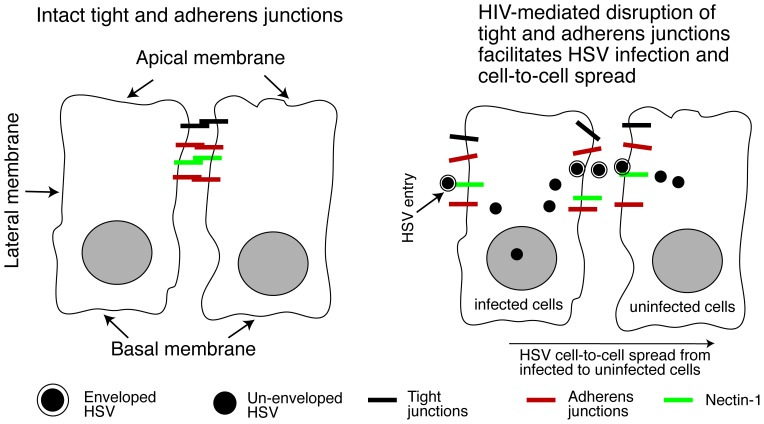
Model of HIV-facilitated HSV infection and spread in the oral mucosal epithelium. The oral mucosal epithelium consists of stratified squamous epithelial cells. Each layer of epithelial cells forms lateral intercellular junctional complexes, including AJs and TJs. HSV-1 gD receptor nectin-1 is sequestered within the intact AJs area of lateral membranes of epithelial cells (left panel). HIV-induced disruption of AJs exposes nectin-1 from its sequestered areas (right panel), which binds to HSV gD and thereby promotes HSV infection and cell-to-cell spread within the oral epithelium. HIV-induced disruption of TJs leads to paracellular spread of HSV virions, which may facilitate penetration of virus from the apical to the basolateral direction into the deeper part of the epithelium, and from the basolateral to the apical direction leading to release of virus into saliva. Thus, HIV-induced disruption of epithelial junctions may facilitate the spread of HSV-1 infection within the mucosal epithelium, leading to the rapid progression of HSV-mediated mucosal lesions and ulcers.

In summary, we have shown that HIV proteins tat and gp120 and cell-free virions disrupt oral epithelial junctions and expose HSV-1 receptor nectin-1, which is sequestered within the AJs. This is critical for infection by HSV-1 and its rapid paracellular and cell-to-cell spread (Fig. 7), suggesting that the interaction of HIV and HSV through the mucosal environment may play an important role in the development of HIV/AIDS-associated HSV disease. Combined with other factors, such as HIV/AIDS-caused reduction of immune response to HSV, this may increase the risk of subsequent development of HSV-associated diseases. Topical inhibitors of HIV tat- and gp120-activated MAPK signaling [21], [22], [23], [24], [25], [26], [128], [129], [130], [131], [132], [133], [134], [135], which plays a key role in disruption of epithelial junctions and exposure of nectin-1 in HIV-infected individuals, may be useful for reducing HSV-1 spread within the oral and genital epithelium.
