# Correction: HIV-Associated Disruption of Tight and Adherens Junctions of Oral Epithelial Cells Facilitates HSV-1 Infection and Spread

**DOI:** 10.1371/journal.pone.0098724

**Published:** 2014-05-21

**Authors:** The PLOS ONE Staff

There are 2 errors in the first paragraph of the Results subsection titled “HIV tat and gp 120 and Cell-free HIV Virions Induce Activation of MAPK Signaling in Polarized Oral Epithelial Cells.” In the first sentence, αϖ should be αv. In the third sentence, ϖ should be αv.

In the Discussion section, there are 2 errors in the fourth sentence of the third paragraph. In the fourth sentence, “ωηιχη” should be “which” and “λεαδing” should be “leading.”
